# Nature Relatedness as an Orientation in Moral Psychology

**DOI:** 10.1007/s12124-026-09996-x

**Published:** 2026-05-05

**Authors:** Joel Janhonen, Juuso Kähönen, Irina Salmi

**Affiliations:** 1 Department of Clinical Medicine, University of Turku, Turku, Finland; 2 Finnish Institute of Bioethics, Tampere, Finland; 3 Department of Philosophy, University of Helsinki, Helsinki, Finland; 4 Finnish Association for Psychedelic Research, Helsinki, Finland; 5 Department of Psychology and Speech-Language Pathology, University of Turku, Turku, Finland; 6 Turku Environmental Ethics Research Center, Turku, Finland

**Keywords:** Moral psychology, Nature relatedness, Biophilia, Ecological self, Moral foundations theory, Moral expansion

## Abstract

In this article, we theoretically explore the role of biophilia for morality, challenging and expanding recent moral psychological models that struggle to account for this relationship. We conceptualize the psychological trait of Nature Relatedness as a comprehensive biophilic orientation or *mode of being* that enables individuals to integrate nature into their identity and informs their interactions with both natural and social environments. By synthesizing theories of Erich Fromm and Arne Næss with recent research, we claim that biophilic orientation involves not only the transition from a *narrow* to an *ecological* self, but also encompasses worldviews, values, and experiences. We develop the idea that dispositional aspects of biophilia and affective-experiential levels of morality interact: Biophilic disposition is both driven by and drives self-transcendent experiences and affect-laden engagements that foster self-other overlap, possibly expanding one’s moral concern. We connect these ideas to moral psychological research on moral intuitions and identity, particularly by discussing Nature Relatedness in relation to Jonathan Haidt’s Moral Foundations Theory. One’s subjective relationship and identification with the natural world appear central to moral expansion. Besides extending concern to non-human individuals, parochial, group-cohesion-related moral emotions might be re-channeled. These *binding foundations* appear generally resistant to moral expansion, yet the biophilic drive to integrate and unite may broaden one’s identification beyond human ingroups. Thus, we propose that Nature Relatedness may expand moral intuitions across foundations, outlining what a non-anthropocentric application of moral foundations and biocentric intuitions might look like. Implications, including prospective interventions, and the need for further research, are discussed.

## Introduction

Amid looming ecological crises, to better understand and accommodate human concern for the biosphere and non-human lifeforms, there is a need to move towards non-anthropocentric morality and to re-evaluate theories of moral psychology accordingly. We propose a framework linking Nature Relatedness (NR), biophilia, and self-expansion to moral intuitions, and suggest extending the Moral Foundations Theory (MFT) toward a biospheric direction.

NR refers to the extent to which human beings feel connected to nature. NR and nature exposure have been widely studied as a resource that predicts greater well-being, prosociality, and pro-environmental behavior (Barragan-Jason et al., 2022, 2023; Capaldi & Zelenski, 2015; Mackay & Schmitt, 2019; Putra et al., 2020; Shuda et al., 2020; Whitburn et al., 2020; Zelenski et al., 2015). Beyond work on prosociality (Goldy & Piff, 2020), little attention has been given to its relationship with moral dispositions – though emerging research suggests NR may influence moral boundaries, awareness, and behavior (Crimston et al., 2016; Drosinou et al., 2023). Similarly, biocentrism is recognized as a distinct value orientation (de Groot & Steg, 2007), yet its psychological mechanisms and links to moral psychology remain unclear. To our knowledge, no comprehensive theoretical attempt has yet bridged NR with psychological moral frameworks.

MFT is a framework in moral psychology that explains cultural variation in moral judgements by positing that moralities are built upon a set of innate, universal foundations such as care, fairness, loyalty, authority, and sanctity. Relevant to our work, it is worth noticing that the MFT primarily focuses on social relationships between people, leaving ethical judgments concerning our relations with non-human species or nature largely untouched. Haidt describes life as a hierarchy of nested levels from genes and cells to organisms, hives, and societies. However, for the purposes of moral psychology, according to Haidt, only the levels of the individual organism and group are relevant (Haidt, 2012, p. 224). This focus reflects the social psychological roots of MFT, which, like many contemporary theories, views morality primarily as an evolutionary and game-theoretical adaptation to the problems of human co-operation (e.g., Curry, 2016; Curry et al., 2019; Rai & Fiske, 2011; Greene, 2015). Consequently, MFT is bound by paradigmatic anthropocentrism and lacks the scope to account for moral relations that extend beyond humankind to the biotic community.

However, morality is often sensitive to the nested structure of life beyond groups of people – expanding to other species, to ecosystems, even to the biosphere. We consider it a major (meta)theoretical limitation if a theory of human morality focuses only on human-human interactions. A comprehensive descriptive theory of morality should be able to accommodate environmental ethics and non-Western indigenous and animist perspectives where morality axiomatically involves respect, care, and similar ways of relating towards non-human entities (e.g., Callicott, 2015; Thiermann & Sheate, 2020; Whyte & Cuomo, 2016; Woodhouse et al., 2021; Paul et al., 2021).

In this paper, we build a theoretical framework to understand the effects of NR on morality and apply this to MFT (Haidt, 2012). We argue that variation in NR and expansion of the self significantly alter moral intuitions, based on how one relates to *others* – boundaries and core assumptions of the *self* moderate whether one is only concerned about their immediate interests, that of a narrow ingroup, or whether concern extends beyond.

The next section of this paper presents NR as a biophilic orientation. We review literature, discuss the theoretical background of NR, and adopt a viewpoint based on Erich Fromm’s (1964) version of biophilia, deepened by Arne Næss’ (1973) self-expansion and the “Ecological Self”. We consider NR (or the lack of it) to be fundamental in how the human organism relates and interacts with its environment. The third section turns to the general relevance of NR for morality. In line with prior literature, we propose that a sense of relatedness is foundational to an inclusive environmental ethic. The fourth section of the paper will attempt to merge NR with MFT, exploring the expansion of its foundations. Rather than only focusing on its influence on environmental attitudes and actions, we explore a more general influence – possibly permeating all our foundational moral drives. Finally, we consider implications of the framework for interventions, limitations, and sketch directions for future research.^1^

## Nature Relatedness as a Biophilic Orientation

### Nature Relatedness

By “nature” we refer to the interrelated web of life and the sustaining abiotic environments within which individual organisms are embedded. Distinctly, NR has been studied by Nisbet and Zelenski (2009, 2013). Their NR questionnaire measures cognitive, affective, and experiential dimensions of the human-nature relationship, with three subsections measuring conceptions of self (or identity), perspectives (or worldviews), and experiential dimensions. In this section, we will elaborate on these essential components of NR.

The questionnaire by Nisbet and Zelenski is not the only psychological measure to study people’s subjective sense of connection to nature (see Tam, 2013). Unlike the NR scale, many scales and constructs concentrate on only some aspects of this multidimensional phenomenon. For example, the Connectedness to Nature scale focuses on feelings of connectedness (Mayer & Frantz, 2004), and Schultz’s (2002) Inclusion of Nature in Self focuses on the cognitive side of NR. These different measures and concepts correlate strongly with one another (Tam, 2013), suggesting that there may be a higher-order construct underlying them. In this paper, we chose to use the term *NR* to refer to this underlying construct because the NR scale encompasses multiple aspects of the phenomenon, and considers the connection to be a relatively stable, multidimensional trait-like orientation rather than a state-like, temporary experience. Keeping in mind that most measures strongly correlate with one another, we primarily use the term NR, and alternatively, the concept of *biophilic orientation* in this work to refer to the *higher-order construct underlying the variety of measures* in Tam’s analysis. With this solution, we emphasize that while temporary experiences are critical, a broader integration involving identity, cognitive, affective, and experiential dimensions is more suitable for understanding the complex relation of NR and moral psychology.

Due to the associations with pro-environmental and prosocial inclinations, NR is relevant to morality in both human and more-than-human domains. While the empirical findings between NR and pro-environmental inclinations are solid, the theoretical framework to explain this connection – and how it links to moral dispositions – remains underdeveloped.

### Biophilia as an Orientation

Theoretically, Nisbet and Zelenski (2013) base their conceptualization underpinning the NR scale on the *biophilia hypothesis* initiated by E.O. Wilson (1984). The biophilia hypothesis suggests that humans have an evolutionarily based innate tendency to focus and affiliate with life and life-like processes, developed to ensure our nature-dependent survival.

As a term, biophilia was first coined by Erich Fromm, although this is seldom acknowledged. The Frommian concept of biophilia (1964) extends it from a useful adaptation of the human species to something concerning all life. He agrees with Wilson’s definition of “love of life” or being attracted to and fascinated by all that is alive. Fromm continues, however, that in humans:

*“Biophilia is not constituted by a single trait*,* but represents a total orientation*,* an entire way of being. It is manifested in a person’s bodily processes*,* in his emotions*,* in his thoughts*,* in his gestures […] The most elementary form of this orientation is expressed in the tendency of all living organisms to live.”* (Fromm, 1964, p. 45).

The fundamentality of biophilic orientation would explain the multidimensionality of NR: It is expressed in the entire way of being, and manifests in domains of cognition, affect, experience, and other dimensions, including those related to morality.

Notably, Fromm’s biophilia includes the tendency to *“integrate and unite with other life”*, in addition to the elementary drive for self-preservation. These ideas of fundamental biophilic orientation in all life were preceded already at the turn of the 20th century by Peter Kropotkin, a natural scientist who, shortly after Darwin’s seminal works on natural selection (in 1902), suggested a parallel force or cooperative strategy he termed “mutual aid”, alongside individualistic struggle for survival. For Kropotkin, mutual aid was to be seen “*not only as an argument in favor of a pre-human origin of moral instincts*,* but also as a law of Nature and a factor of evolution*”. (Kropotkin, 1987, introduction.) Congruently, contemporary *systems biology* sees that all life shares basic tendencies toward autonomy/self-sustenance and integration (Boogerd et al., 2007; Moreno & Mossio, 2015).

These tendencies might motivate mutualism and cooperation in the form of a meta-motivational bias – or a relational orientation – manifested species-specifically to match the niches of each living creature, ranging from simple lifeforms such as bacteria to primates (de Waal, 1996; de Waal, 2008; Damasio, 2018). Such a bias may also be ingrained in key human drives, fostering cooperation and care both within kin as well as towards the more-than-human world.^2^

Assuming the existence of such a biophilic orientation necessitates accommodation of moral psychological theories. Jonathan Haidt argues that while much of human behavior can be explained through “enlightened self-interest,” humans are also inherently “groupish”, possessing psychological systems promoting the group’s interests in competition with others (Haidt, 2012). It could be argued that humans, in addition to being groupish, are inherently *relationally biocentric*, possessing mental proclivities and mechanisms for promoting the interests of ecosystems and the whole web of life, and are therefore able to operate on a moral level that transcends the boundary of the human group – much as the group-boundary transcends the self-boundary.

Biophilic orientation consists of interlinked, mutually reinforcing dimensions that vary in emphasis across individuals and groups. Relatedly, we next examine: (1) worldviews and values, (2) ecological identity and self-expansion, and (3) dispositions for self-transcendent experiences (STEs) and emotions.

### Holistic Worldviews and Sustainability-Aligned Values

Culturally transmitted worldviews, beliefs, and values are central to manifesting biophilic orientation. Worldviews shape our ontological understanding of the self, humanity, and the non-human world, while values imbue these beliefs with normativity, such that evaluative assumptions embedded in the context are internalized and come to prime our judgments. NR subscale on perspective includes elements of both (Nisbet et al., 2009).

Richard Nisbett, with his colleagues (2001), emphasize how holistic versus analytic thought systems profoundly shape what Fromm called our “entire ways of being.” Some varieties of holistic or relational worldviews foster interconnectedness beyond the human ingroup, such as animistic and indigenous worldviews and Eastern philosophies (e.g., Harvey, 2005; Macy, 1991). By contrast, the dominant Western scientific worldview has been anthropocentric, atomistic, and individualistic, fostering instrumental attitudes and alienation from nature (Beery et al., 2023; Bateson, 2002).^3^ Yet, holistic currents within the West – e.g., deep ecology, systems thinking, and neo-materialist philosophies – also promote biophilic viewpoints (Vetlesen, 2019).

Values, as integral components of worldviews (Koltko-Rivera, 2004), represent individuals’ most general goals, guiding moral attitudes and behavior. Research on pro-environmental values distinguishes egoistic, altruistic, and biocentric orientations (de Groot & Steg, 2007, 2010). More recently, sustainability-aligned values have been proposed to encompass altruistic, relational, and self-transcendent orientations that correlate with and promote environmentalism (Martin et al., 2024; Buijs et al., 2025; Himes et al., 2024).

Empirical measurements of NR correlate with environmental values and have been proposed to be both a measure and a causal factor underpinning them (Lengieza & Aviste, 2025; Martin & Czellar, 2017; Dutcher et al., 2007; Restall & Conrad, 2015), and may also mediate the link between environmental values and behavior (Pereira & Forster, 2015). This entanglement of NR and values forms a key normative dimension of biophilia, as pro-environmental or biospheric values are major antecedents of pro-environmental behavior (Wang et al., 2021; Tamar et al., 2021; de Groot & Thøgersen, 2018).

### Ecological Self and Identities

Another key factor – closely related to one’s worldview and values – in NR is identity and the sense of self. The NR scale includes items on self-nature overlap as well as identity (Nisbet et al., 2009). To clarify these notoriously ambiguous concepts (Thagard & Wood, 2015), by *self* we refer to the defining core of selfhood, encompassing both experiential (sense of self) and ontological (what I am) dimensions, while by *identity*, the narrative and social aspect of self, how one sees oneself through socially embedded self-definitions. However, these intertwine and are sometimes used synonymously in the literature.^4^

NR is closely linked to the expansion of self and identity. The most influential theoretical formulation of this phenomenon is Arne Næss’s (1987) concept of the *Ecological Self*, which was inspired by Fromm’s work. Næss, through his distinction between the “narrow self” and the “Ecological Self”, can be understood to highlight a continuum within biophilia: the extent to which one focuses on one’s personal benefit and self-sustenance versus identifies as, and is concerned about, the whole “web of life”. Næss saw that identification with all of nature was the foundation of environmental concern. Subsequent theoretical work has refined this notion. Bragg (1996) develops the theoretical foundations of identification from the perspective of constructionist self-theory, suggesting Ecological Self as an extension of *interdependent self*, while casting doubt on whether fully encompassing Ecological Self can be sustained in daily life, in contrast to temporary experiences of identification. Viewing narrow and Ecological selves as extremes of a situation-sensitive, responsive continuum rather than as either-or features might be one means by which to alleviate this tension. Similarly, while environmental identity is conceptualized as a stable individual feature that predicts behavior (Clayton, 2003), it remains subject to the situational fluidity inherent in all identity constructs, activated and becoming salient when cued by the immediate context (Burke & Stets, 2009). This is echoed by Macy (in Edelglass, 2009), who discusses the Ecological Self in the context of Buddhist thought and systems thinking – defining the self not as a bounded entity but as a process of interconnections.

The idea that our self-constructs can widen towards ecological inclusion gains support from empirical studies. The NR scale and related measures show correlations between biophilic self-conceptions and pro-environmental values, behaviors, and self-transcendence (Schultz, 2001, 2002; Schultz & Zelezny, 1999; Mayer & Frantz, 2004). Connectedness to nature predicts environmental values and rests on the inclusion of nature within the self (Dutcher et al., 2007). McConnell and Jacobs found that experimentally increasing relative nature–self size enhanced pro-environmental behavior and motivation, and predicted connectedness to nature, biospheric concern, and self-reported pro-environmental behavior (2022; 2020).

While instances of strong ecological selfhood are fleeting, a more psychologically realistic and stable outcome is the creation of environmental social identities (Clayton, 2003). In a meta-analysis, Lou and Li (2021) found that various measures of environmental identity showed moderate-to-large effects on environmental concern, moderated by societal collectivism versus individualism (i.e., stronger effects in individualistic societies; see also Chan, 2020). Similarly, Lokhorst et al. (2014) found that self-identity (e.g., identifying as a conservationist or environmentalist) mediated the link between connectedness to nature and conservation intentions.

Research also indicates that identities mediate the link between environmental values and behavior. Values lie at the core of one’s identity (Hitlin, 2003). In environmental science, a value–identity–behavior pathway has been identified, where biospheric values predict environmental identity, which in turn shapes preferences, intentions, and behaviors (van der Werff et al. 2013a, b; Wang et al., 2021). Similarly, Gatersleben et al. (2012) found that values and identities, rather than attitudes, predicted pro-environmental behavior, with the effect of values fully mediated by identity. These findings also illuminate the “value–action gap,” the discrepancy between self-reported pro-environmental values and behavior (Kollmuss & Agyeman, 2002). For instance, after value activation, only values central to one’s identity influenced behavior, highlighting the role of identity in translating values into action (Verplanken & Holland, 2002).

### Connecting to Nature through Self-Transcendent Experiences and Emotions

A third major factor shaping biocentric orientation is individuals’ experiences, particularly self-transcendent varieties, which can be considered as significant experiential-epistemic factors underpinning biophilia. In the NR sub-scales, NR-affect and NR-self items exhibit self-transcendent experiential and affective features (Nisbet et al., 2009). In self-transcendent experiences (STEs), (1) the salience attributed to the individual self is diminished, and (2) an experiential overlap occurs between self and other (Yaden et al., 2017). A particularly relevant subtype of STEs is self-transcendent positive emotions (STPEs) – other-focused emotions that broaden attention beyond oneself, such as awe, gratitude, compassion, love, humility, and reverence (Jacobs & McConnell, 2022). These experiences can be studied as dispositions (tendencies for recurrent experiences) or states (discrete events). Dispositions are more central to biophilic orientation, though sometimes even single STEs may influence biophilic tendencies.

STEs have been proposed to foster cooperation (Pizarro et al., 2021, 2022; Stellar et al., 2017). Haidt links self-transcendent emotions, especially awe, to the so-called “hive switch” – a supposed group-oriented adaptation that shifts thinking and behavior from individualistic to groupish by diminishing the self and making one feel as “a part of a whole” (Haidt, 2012, p. 264). However, we propose that the “hive switch” or self-transcendence can be directly triggered by – and align us with – wider than human ingroup wholes, rather than it being a “side-effect” of a mechanism related only to the human ingroup. STEs in relation to nature or the more-than-human world diminish the (narrow) self, and can make us orient *groupishly* biocentrically, beyond human ingroups. However, the occurrence and outcomes of STEs in any given situation depend on factors such as the breadth of one’s default identification, values, and personality.

Research supports the biophilic effects of STEs and STPEs. STEs enhance nature connectedness, pro-environmental behavior, and ecological well-being (Isham et al., 2022). Dispositions toward awe, compassion, and love predict greater pro-environmental behavior and biocentric concern (Jacobs & McConnell, 2022; Zelenski & Desrochers, 2021). Trait mindfulness, overlapping with self-transcendence, also correlates with nature connectedness (Schutte & Malouff, 2018; Barbir et al., 2025). Thus, experiential strategies that foster connectedness and empathic care for nature have been proposed as a method to foster pro-environmental behavior (Thiermann & Sheate, 2020).

Nature-based experiences can promote altruism, cooperation, and environmental behavior (Zelenski et al., 2015; Barragan-Jason et al., 2023; Liu et al., 2022), and STEs may be one underpinning mechanism (Lumber et al., 2017; Castelo et al., 2021). Immersion in nature often leads to profound experiences of unity with the natural world (McDonald et al., 2009; Aaltola, 2015). Studies show that STPEs lead to increases in scores of the NR scale, while moral elevation and gratitude enhance NR via STPEs (Moreton et al., 2019; Chen et al., 2022). Similarly, compassion fosters pro-environmental tendencies and intentions (Pfattheicher et al., 2015), while NR is positively associated with empathy and inversely with callous–uncaring traits (Fido & Richardson, 2019).

STEs help explain variation in the self-nature overlap, as self-other overlap defines STEs (Yaden et al., 2017). Awe diminishes individual self-focus and promotes collective engagement (Piff et al., 2015; Bai et al., 2017). STPEs predict the inclusion of nature in self, biospheric concern, and pro-environmental behavior (Jacobs & McConnell, 2022; McConnell & Jacobs, 2020), arguably providing temporary shifts toward the Ecological Self (cf. Bragg, 1996). Recent psychedelic research supports the notion that STEs can lead to increased NR, pro-environmental behavior, as well as to other related outcomes such as self-transcendent values, moral expansiveness, and solidarity towards animals (Irvine et al., 2023; Kettner et al., 2019; Nilsson & Stålhammar, 2024; Forstmann & Sagioglou, 2025; Newton & Moreton, 2023 Kähönen, 2023; Olteanu & Moreton, 2025; Pöllänen et al., 2022).

## Moral Relevance of Nature Relatedness and Biophilia

### Empirical Findings: NR Affects Moral Expansion and Awareness

The studies reviewed above regarding how NR relates to environmentalism suggest that this orientation may have moral relevance. Next, we elaborate on its moral significance by exploring its potential as a catalyst for *moral expansion*. Peter Singer (1981) envisioned in his account of the *Expanding Circle* that individuals’ and whole societies’ moral consideration can widen, coming to include non-human entities. However, meaningful moral concern requires not only the circle’s breadth – who or what is deemed morally relevant – but also depth: the intensity of concern allocated. Crimston et al. (2016, 2018) found that moral expansiveness – measured by the breadth and depth of concern for entities including plants, animals, and the environment – correlates positively with connectedness to nature and universalism values, and predicts prioritization of humanitarian and environmental concerns over self-interest and willingness to act sacrificially.

Similarly, Drosinou et al. (2023) report that awareness of self, others, and nature correlates with moral awareness of environmental protection. Moral awareness predicted environmental concern and behavior more strongly than connectedness to nature, which nonetheless outperformed self- or *other-awareness*. These findings suggest a mutually reinforcing relationship between awareness of nature and moral awareness. However, both studies used the one-item Inclusion of Nature in Self scale – a narrower construct than the NR scale.

While research specifically linking NR and morality is currently limited, its influence on moral judgment is being investigated (Janhonen et al., 2024). Preliminary data from the Finnish Institute of Bioethics revealed a positive association between the NR scale and the perceived moral significance of dilemma scenarios (MyBioethics, 2025), suggesting that it may broadly influence moral perception and decision-making.

### Biophilic Moral Concern and the Worldview-identity-value-nexus

As a plausible theoretical hypothesis, NR encompasses ontological (conceptions of self and world), epistemic (ways of knowing and paying attention), and normative dimensions (attitudes, attributions of value, and sense of moral salience), each affecting morality.

The ontological dimensions in one’s conception of self and world are morally relevant. Consider this quote from Warwick Fox, which suggests a link from the ontological level to the normative:

*“The deep ecologists’ analysis of the self is such that they consider that if one has a deep understanding of the way things are (i.e. if one emphatically incorporates the fact that we and all other entities are aspects of a single unfolding reality) then one will (as opposed to should) naturally be inclined to care for the unfolding of the world in all its aspects.”* (Fox, 1995, p. 247; Quoted in Aaltola, 2015).

A more inclusive identity expands moral concern by incorporating the interests of a broader range of beings. Just as interpersonal closeness involves including others in one’s self-concept (Aron et al., 1992), merging self and nature extends goals and concerns to non-humans (Schultz, 2001; Lengieza, 2024).

Moral concern depends not only on self-conceptions but on how reality is understood. Whether our standpoint is relational or biocentric largely reflects our worldviews. For example, attributing experiential capacities such as pain or fear to animals influences moral judgments and justifications of violence (Potocka & Bielecki, 2023). Both empirical knowledge and worldview shape imaginaries that (dis)enable perspective-taking and concern beyond human contexts (e.g., awareness of factory farming and belief in animal sentience).

Identities and worldviews are further intertwined with values and affective dispositions. Vetlesen (2017) criticizes Næss for neglecting relationality, inherent values, and otherness in his Ecological Self. What may matter most is not merely expanding self but expanding fundamental concern – though these often coincide.

Biophilic moral sensibilities seem to involve a responsiveness to the intrinsic and relational value of the non-human world. Non-egocentric, non-anthropocentric dispositions to extend care and attentive concern to the non-human world (cf. Aaltola, 2022) involve recognizing the intrinsic value of more-than-human beings while responding to their flourishing and suffering – that is, perceiving the non-human realm as salient and morally significant.^5^ What is important is not only abstract expansion of moral concern, but how biocentric moral considerations concretely weigh against self-enhancement and anthropocentric moral goals and guide our attention and decisions, as manifested in modes of attention and affective tendencies.

Thus, in contrast to early deep ecologists like Næss, who emphasized self-expansion, we propose that expansion of moral concern arises from a biophilic nexus of worldviews, values, identities, and experiential/affective tendencies. These factors shape the boundaries of what and who is considered morally salient, often preceding conscious deliberation. Feeling connected to other forms of life and relatedness across species is in reciprocal relation to extending moral concern beyond oneself and the ingroup. Thus, biophilic orientation may be considered as central for broadening moral regard: a different mode of being and relating brings about a different mode of moral appraisal.

However, increased NR may not automatically lead to pro-environmental ethical judgments and ecological behavior. There likely exist counterexamples of persons who have relatively high NR but still engage in a variety of harm-producing actions towards non-humans and nature, and possibly persons who lack some aspects of NR (e.g., experiential and affective connection) but still exhibit moral concern and obligation towards the non-human world. As argued, NR itself consists of multiple interlinked factors, and perhaps some configurations make a greater moral difference, which is a central question for future empirical research. The expansion of the moral circle is not linear, and which entities receive moral concern depends on multiple factors, such as identity processes, substantive beliefs, and situational factors (Crimston et al., 2016, 2018).

### Emotions and Epiphanies as Sources of Moral Intuitions and Knowledge

**Fig. 1:**
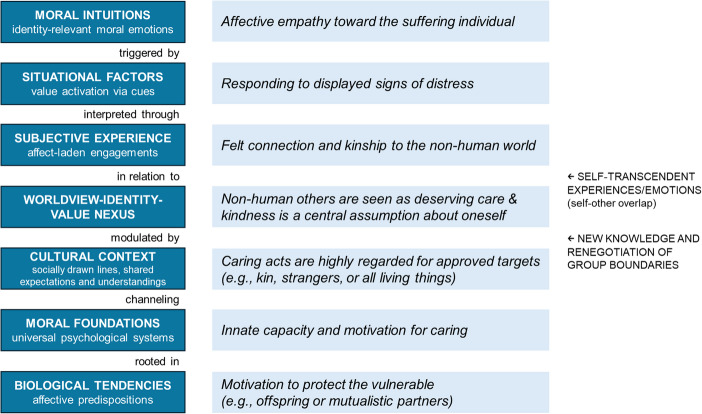
Relations between analyzed concepts from biological predispositions to affective reactions. Different levels of moral cognition are illustrated with the care/harm foundation expressed through empathic concern

To further clarify how NR relates to morality, we propose a way to understand how selfhood, values, emotions, and moral intuitions are intrinsically connected (Fig. 1). This account can explain the moral role of biophilic emotions while preserving their epistemic value – by positing them as arising from a grasp and recognition of the value of the non-human world instead of mere pre-rational gut reactions supposed by the social intuitionist model (cf. Kauppinen, 2015).

In recent moral philosophy, the epistemic role of experience and emotions has been explored (for broader discussion on the relation between emotions and morality, see Prinz 2009; Kauppinen 2024a). In line with Haidt’s theories, recent philosophical sentimentalism holds that moral intuitions are fundamentally sentiments – dispositional tendencies to feel, manifested as emotional reactions (Kauppinen 2013, 2024a). In a slightly more delineated sense, while exploring the relation between values, selfhood, and emotions, Monticelli (2018) defines sentiments as *identity-relevant emotions* (in contrast to situational affective states such as hunger or amusement). We suggest that biophilic orientation shapes moral intuitions by expanding the scope of identity-relevant emotions. As identity and values broaden, so do moral emotions – extending concern, approval, and disapproval to matters involving nature, now rendered salient for the self.

Empirical research on eco-emotions supports this view. For instance, Ágoston et al. (2024) found that the link between the NR scale and pro-environmental behavior was mediated by eco-emotions such as grief and guilt – sentiments arguably arising from threats to an identity-relevant and valued natural world. Likewise, the positive eco-emotions and STPEs can be seen as affective manifestations of the worldview-identity-value nexus that underlies and feeds into our moral motivation and intuitions. For instance, Jacobs and McConnells (2022) found correlations between STPEs and individuals’ value orientations. Together, these findings suggest that both positive and negative eco-emotions are identity-relevant affects, responsive to core valuations, further shaping our caring responses and behavior.

Furthermore, some of these emotions, such as empathy and awe, can be understood as epistemologically important moral emotions in the context of biophilic orientation. Many philosophers suggest that emotions are ways of apprehending values: we perceive danger through fear, and positive value(s) through emotions such as desire, affection, or respect (Kauppinen 2024b; Goldie 2000). In this vein, both eco-emotions, surveyed above epiphanic experiences in nature, and empathy towards non-human others can epistemically attune us to the value of the non-human world (Aaltola, 2015, 2022, 2025). STEs, emotions, and imaginative role-taking can thus be seen as a central epistemic route for biophilic moral expansion, and factors underpinning our moral intuitions, as they bring us into contact with relevant biophilic values.

Moral expansion often arises through messy, embodied, affectively charged experiences that foster care and self-other overlap, deserving attention alongside conscious, principled decisions. While we have emphasized NR’s moral-epistemic relevance via affective processes, rational and deliberative aspects remain important. Greater relatedness does not replace reasoned compassion or codified ethical principles; rather, affective and rational mechanisms can mutually support each other. Both analytical reflection and intuition are central in moral decision-making and can prime and modulate each other. Thus, we echo Aaltola (2022) in viewing emotional capacities and reason as mutually interdependent components of morality.

## A Proposal for Expanding the Moral Foundations Theory

To explore the sway of NR on moral judgements and decision-making, we focus on the social intuitionist approach to moral psychology (Haidt, 2003). Accordingly, moral stances are primarily products of intuitive processing and emotional dispositions, which are subsequently rationalized and refined through analytical reasoning.

### Moral Foundations Theory

Aligned with the intuitionist view of ethics, MFT is a prominent and comprehensive model for explaining the psychology of morality, suggesting that human moral intuitions are based on universal foundations that are emphasized differently by individuals due to their cultural background and personality.^6^ MFT initially proposed four moral modules with natural triggers and characteristic emotions (Haidt & Joseph, 2004)​, later expanded to six: Care, Loyalty, Authority, Purity, Equality, and Proportionality (Haidt, 2012; Atari et al., 2023), and other candidate foundations have been suggested (MoralFoundations.org, 2024)​​. These foundations of moral intuition have been grouped into individualizing modules, which focus on individual needs and autonomy (care, fairness, liberty), and binding modules, which prioritize the good of the group (loyalty, purity, authority, honor, ownership).​ (Graham et al., 2009)​. One’s location on the individualizing-binding spectrum is expected to account for much of the variation in moral concerns. In general, conservatives place more equal weight on both groups, while liberals center the individualizing set.

As stated, MFT may be unduly constrained by an anthropocentric focus and overlooks non-anthropocentric elements of human morality. Next, we explore existing research and theories that support our proposal to expand this framework.

### Prior Research on Biocentrism and Moral Foundations

Regarding individualizing foundations, ***care*** and ***fairness*** are shown to intensify environmentalism and attenuate anti-environmentalism even among those whose political ideology or social identity might otherwise predispose them against such positions (Milfont et al., 2019). Liberals typically give more weight to non-human *rings* of the moral circle through universalist compassion (Waytz et al., 2019).

Moral expansiveness, in turn, correlates negatively with binding foundations (Crimston et al., 2016), the exclusionary nature of which commonly undermines pro-environmental actions (Milfont et al., 2019). Moreover, conservative ideological orientations predict less biospheric concern and reduced inclusion of nature in the self. This is often characterized by a perceived psychological prominence of the self over nature, a lower endorsement of self-transcendent values, and less pro-environmental behavior, as well as greater egoistic concern and endorsement of self-enhancement values (Jacobs & McConnell, 2022). Relatedly, Ertör-Akyazi and Akçay (2021) studied whether participants prioritized private earnings over pooled earning potential in a climate commons game related to natural resources, but with no non-human beneficiaries: They observed higher care predicting less extraction, higher loyalty predicting more, and purity/sanctity having no effect.

On the other hand, emotional processing and intuitions underlying biocentric concerns appear to cut through the liberal-conservative divide: Psychological work on biocentric attitudes distinguishes at least two types of concern, attitudes based on ***care/harm*** and ***purity/sanctity*** (Rottman, 2014)​. Care/harm generally needs to target an individual – someone to care for – while sanctity or purity are expressed in a more diffused manner (Rottman et al., 2015)​. Among conservatives, environmental messages framed to evoke sanctity and purity concerns appear to work best (Feinberg & Willer, 2013)​​. It appears feasible to co-opt binding foundations to activate environmental concern to turn an individual biocentrically groupish. Ertör-Akyazi and Akçay (2021) emphasize the power of framing when interpreting their results, suggesting that loyalty led to greater extraction, especially because it was evoked in the study through national pride associated with the protection of the ingroup’s material interest over environmentalism.

A more biophilic form of ***loyalty*** may be reflected in various types of allegiances toward ingroups involving non-human others as well as towards Nature itself. While social group memberships partly form our self-concepts, crucially, such categories need not consist merely of humans. When entities are recategorized under a shared superordinate group, ingroup-favoring biases are reconfigured, which allows for broader concern and collaboration (Gaertner & Dovidio, 2000)​​. Moral-intuitive machinery can be extended to care about non-human nature (Clayton, 2003).​ For example, in many indigenous communities, nature is part of the social structure, and from this relational ontology, moral obligations flow (Celidwen & Keltner, 2023).

Loyalty is strongly parochial: As the relevant groups have historically expanded – from tribes to nations – related intuitions may not have expanded accordingly. We suggest that social sorting constrains loyalty’s sphere: Yet, it is just because the line between “us” and “them” is socially drawn, that even a river, forest, or a mountain can be regarded as a member of the community (New Zealand has, during the past 15 years, also done these recognitions legally). Such sorting also operates within human-animal relations: Pets, companions, or working animals that are granted socially recognized roles as members of the ingroup are routinely accorded heightened moral concern, precisely because they are perceived as part of us (Serpell, 1986). Group memberships are overlapping and nested (Kahn, 2022)​. Already, the Land Ethic of Aldo Leopold argues that a good steward of the environment includes the biotic community – soils, waters, plants, animals – in their moral community (1949)​​. Arguably, people tend to feel rootedness, a sense of stewardship and loyalty, especially towards their dwelling places, which, especially before industrialization, were the main sources of sustenance.

Biocentric loyalty may also be expressed by being drawn to – and consistently engaging with pro-environmental (in)groups, motivating self-sacrifice and norm enforcement. However, following Næss (1973) and Clayton (2003), a shallow form of instrumental ecology of ingroup belonging should be distinguished from true self-expansive ecological identification. Biocentric loyalty goes beyond extrinsic motivations.

Haidt himself suggests that the ***sanctity*** foundation often underlies the moral passions of people joining environmental movements. Environmentalists commonly rail against industrialism and capitalism, not only for the concrete degradation of nature but for its more symbolic corruption and defilement (Haidt, 2012, p. 176)​. Such concern often gets expressed in quasi-religious language. Furthermore, explorative research has indicated that NR may affect decision-making when an act is considered unnatural or as a violation of the natural order. Greater sensitivity to experience disgust regarding ethical dilemmas with an *unnatural feel* (van Leeuwen et al., 2017), such as cloning or the legalized sale of non-vital organs (Janhonen et al., 2024), may mark another link between NR and sanctity​​. In sum, biocentric concern appears to draw on diverse moral foundations. Therefore, following Rottman et al. (2014), we suggest that moral expansion is not reducible to care or individualizing intuitions alone.^7^

What people think about the experiences of animals seems to mediate how moral foundations shape decisions about animals, like in cases of instrumental violence, such as in the study by Potocka (2022). It found that care predictably correlated with the rejection of violence, and high authority predicted the acceptance of, or participation in it, likely as animals were seen as subordinates within a human-centered hierarchy. The key insight is that mind perception seems to actively expand the attribution of moral status by broadening the scope of moral concern for an individual. Conversely, the expansion of binding foundations might rely more on redrawing moral delineations. In addition, people vary in their willingness to imagine others’ minds, which in turn predicts the breadth of their moral concern.

### A Proposal: Biophilia Cutting Across Moral Foundations

To reconcile the excessively anthropocentric bias of MFT, we suggest that incorporating biophilic orientation provides an avenue for theory development. We suggest that positing a biocentric scope of moral functioning can meaningfully bridge the ideas of Haidt, Næss, and Fromm, offering a more integrated and insightful understanding of morality. Instead of broadening any single foundation or formulating a new one, we proposed that biophilic meta-orientation may affect all moral foundations (see Table 1).

One route for NR to cut across the moral foundations might be through capturing how central nature is to one’s self, identity, and values. In practice, this cut-through could mean that for individualizing foundations, concern would extend to new kinds of individuals, while binding foundations would be concerned with more diverse groups or coalitions of life. It has been suggested that, originally, the empathic response underlying the care-foundation was narrowly focused on offspring, thereafter extended to kin, ingroup, nation, humanity, and ultimately all sentient beings through rational generalization of emotional responses (Singer, 1981). While moral gains have undoubtedly been attained by, e.g., reasoning about animals’ capability to suffer (Bloom, 2010), motivation to extend consideration may be more about how we relate than how we reason.

Diverse emotional pathways may be involved in the biophilic expansion of moral foundations. As Jacobs and McConnell (2022) point out, Haidt’s moral intuitionism is consistent with emotions’ ability to change values, and that, according to MFT, moral emotions (including STEs) are crucial in developing the moral palate. Haidt has categorized the foundation-relevant emotions into other-condemning, self-conscious, other-suffering, and other-praising (Haidt, 2003). For instance, the experience of awe has been hypothesized to underpin social hierarchies by evoking deference and perceived legitimacy toward social hierarchies and collective values – hence being associated with the ***authority*** foundation (Keltner & Haidt, 2003). However, wondering over the perceived vastness of nature can also challenge existing mental schemas. For example, the cosmic grandeur of the night sky can diminish the perceived significance of oneself, or that of any human, through a profound experience of awe. According to this view, the same moral emotion that narrowly binds us to parental authorities and societal order can be expanded toward the cosmos, as deference to the ultimate order.

In Table 1, we propose a scope-dependent expression of moral foundations. The aim is not to claim exact behavioral outcomes but to show how self-expansion might channel these concerns differently.

**Table 1:** Proposed narrow and broad scopes of the five foundations of the original questionnaire

Foundation^a^	Care	Fairness^b^	Loyalty	Authority	Sanctity
Characteristic emotions	Compassion (empathic concern)	Anger, Gratitude, Guilt (reciprocal altruism)	Group pride, Rage at traitors	Respect, Fear, Awe	Disgust, sacredness
Narrow scope/self	One’s child	Cooperation; cheaters and free-riders	Family, kin, or ingroup	Obedience to superiors	Private/group sexual purity
Broad ecological scope/self	Any sentient being	Ecological injustices, mutualism	Allegiance to nature and life; Rootedness to place	Honoring nature’s autonomy; Humility towards nature; Stewardship^c^	Nature as sacred; Natural order
Case example	Suffering caused by animal testing	The injustice of mass extinction and habitat loss for other species	Inclusion of non-human entities as members; Environmental activism	Living according to nature’s patterns, cycles, and signs; knowing one’s proper place (versus hybris), mimicking nature	Resistance to degrading or unnatural acts like cloning; Reverence for nature and life
Emphasis	**Individualizing**		**Binding**		

a The later liberty/oppression foundation, for opposing bullies, might also map onto the proposed ecological scope, e.g., with notions of self-willed nature and calls for rewilding

^b^ Anger toward cheaters can also include more binding proportionality concerns. The original fairness foundation bundled (at least) two distinct types of distributive justice motives and was later split into equality (individualizing) and proportionality (binding) foundations (Atari et al., 2023)

^c^ Conception of stewardship as a delegated duty; the individual acts not as an owner but as a trustee obeying a mandate from a higher authority, e.g., God(s), ancestors, or future generations

## Implications and Discussion

### Prescriptions and Interventions

If biophilic orientation/NR is found to be a factor closely related to morality and predictive of moral development, as described in this paper, the potential for its intentional cultivation warrants careful consideration. NR appears to sit about halfway between stability and malleability – durable enough to predict behaviors but plastic enough to be momentarily altered or longitudinally shifted through repeated engagement (Sheffield et al., 2022). Sustained change in it may require restructuring of core assumptions, which can be attempted through practices such as mindfulness (Schutte & Malouff, 2018) or, relatedly, the structured use of psychedelic compounds (Kettner et al., 2019; Nilsson & Stålhammar, 2024). Softer methods cultivating a range of moral emotions could be harnessed to support one’s moral life by deepening and defending one’s attachment to nature (Lumber et al., 2017).

Quite obviously, nature immersion may be one method of fostering biophilic orientation. Neurological evidence suggests that immersion in nature reduces the brain’s self-referential processing, thereby broadening focus from repetitive self-rumination (Bratman et al., 2015). This aligns with the philosophical vision of deep ecology, in which the self becomes nested in broader ecological wholes. Exposure to natural environments also appears to automatically induce behavioral changes in a pro-environmental direction, via both psychological and physiological reactions (van den Bosch & Depledge, 2015). Previous research has shown that while nature exposure directly has several positive outcomes for our well-being and behavior, the subjective sense of connectedness or relatedness may enhance these benefits – and influence us also independently of physical contact with nature (Barragan-Jason et al., 2023; Chang et al., 2024; Liu et al., 2022). Thus, even before a more complete picture of NR’s influence on moral psychology is developed, methods to elevate it are well justified based on its well-being benefits, which, if our framework is on the right track, may also lead to additional ethical outcomes.

If NR is central to morality, this implies that the interplay of education and socialization towards NR and biophilia deserves increased attention. While young children often have a deep connection with nature (Barrable & Booth, 2020), this seems to attenuate during adolescence. Maintenance strategies to preserve this connection have been approached from several viewpoints during recent years (see, for instance, Barrable 2019; Giusti et al. 2018; Salmi et al. 2024; Salmi 2025a). The decline of traditional human practices involving direct interaction with nature, like farming and foraging, disrupts the intergenerational transmission of relational values (Chan et al., 2016). This necessitates research into how modern leisure-based engagements can effectively substitute for the sense of stewardship and embodied interaction with nature that traditional practices once provided. Without a deliberate investment in the human-nature relationship, modern culture may struggle to effectively activate our inherent biophilic potential.

### Limitations and Future Research

This work has been largely a descriptive exploration of the role that NR may play in our moral lives. We do not offer any blanket normative endorsement of the biophilic orientation. NR may also raise potential worries, including overlooking more immediate or approximate needs, confused allocation of concern (unwarranted over-attribution of moral status), or idealizing the natural state by projecting inherent purposes or meanings onto it. Also, if the expansion of moral foundations extends considerations of authority or sanctity to non-human nature, one could become predisposed to conflate natural with good, i.e., committing naturalistic fallacy.

Because our aim was to synthesize conceptual and thematic connections rather than evaluate the quality of evidence, we did not apply standardized study selection or appraisal tools typically used in systematic reviews (e.g., PRISMA-based criteria). This limits the review’s capacity to provide a comprehensive overview of the research fields in question and to assess the methodological robustness of the included studies. Although our integrative review approach allowed us to combine studies with heterogeneous methodologies, conceptualization, and paradigmatic backgrounds, which broadened the scope of the synthesis, it also introduced uncertainties regarding comparability. Moreover, study selection was guided by our interest in exploring literature that aligns with our proposed synthesis, which may be a source of bias. In the future, systematic reviews are needed to establish a more methodologically robust picture of the existing research landscape.

Investigating how differences in how we connect and relate to the natural world influence individuals’ moral choices and attitudes provides an interesting new avenue for empirical research. We join Rottman and colleagues (2015) in encouraging research on moral cognitive methods that reliably evoke appropriate concern for non-human beings and make non-human interests more salient. However, besides rational reframing, these should include engagements and experiences that establish affectionate bonds and broaden moral intuitions. Furthermore, models of behavioral economics should not consider people as purely self-interested but acknowledge the intrinsic motivations for prosocial and pro-climate behaviors (Ertör-Akyazi and Akçay 2021; Thiermann & Sheate, 2020).

Whether high-NR individuals score differently on moral foundations or whether NR moderates the scope rather than increases endorsement of specific foundations should be further studied. Conceptual bridging of environmental and moral psychology using work on moral self-expansion and identity is a rich terrain for empirical investigation. Applying NR measures in research on decision-making may help further explain variations in moral intuitions across individuals and groups despite shared foundations.

Research on the associations between NR and moral foundations should account for cultural variability. Work on NR has, until lately, suffered from a Western-bias, but this shortcoming was recently addressed by Barbett and colleagues (2026). Their large-scale survey study consisted of 36,803 participants in 75 countries. The NR scale was found to positively predict various well-being outcomes across countries. Associations were stronger in collectivist-oriented countries compared to individualist-oriented ones. As the collectivist-individualist axis also influences moral foundations by prioritizing either binding or individualizing foundations (Graham et al., 2011), the interactions between these phenomena should be considered, which would further explicate the processes underlying the moral relevance of relatedness.

Similarly, more research is needed on the moral significance of STEs. Jacobs and McConnell (2022) point out that both MFT and the social intuitionist model of morality, as two leading frameworks in moral psychology, effectively support the possibility that STEs influence moral functioning. Detailing the effects of contemplative practices, such as meditation, on aspects of morality (Berryman et al., 2023) – including intuitions and identity – could be directly transposed to measure the effects of NR on these same dimensions.

## Concluding Remarks

This article outlined how Nature Relatedness (NR) can be conceptualized as a biophilic orientation that affects the scope of moral psychology, demonstrating the need to update current psychological models such as the Moral Foundations Theory (MFT).

Through a Frommian reading of Arne Næss’s Ecological Self, we understood NR as an existential orientation that extends care and concern towards non-human life. We proposed that NR, as a biophilic orientation, is constituted by an interrelated, mutually arising nexus of worldview, values, selfhood, identity, and involves self-transcendent experiential and affective dispositions. We argued that this biophilic expansion of the worldview-value-identity nexus facilitates a biophilic widening of moral concern. We offered a sentimentalist account that posits eco-emotions, empathy, and self-transcendent experiences (STEs) as epistemic grounds for biophilic moral intuitions.

Congruently, we argued that MFT should be updated to encompass morality towards other-than-human beings and collectives. We proposed that the boundary of moral intuitions is linked to how an individual relates to non-human others and ecosystems. We argued that a biophilic orientation may cut across multiple distinct evolved psychological systems that generate moral intuitions and judgments. We showed how each moral foundation and the intuitions central to MFT could be extended to explain and predict non-anthropocentric moral responses. Our proposal offers a way to understand NR’s broad, tentative influence on personal ethics and expand MFT beyond the levels of the human individual and group.

Through biophilia, we can investigate whether human psychological modules posited by MFT can motivate the preservation and sustenance of interconnected life, thereby manifesting biocentrical groupishness and the concern for interests beyond family or social relations. Instead of romanticizing nature, biophilic orientation can be understood as a morally relevant trait, foundational for respect, care, and collaboration with the more-than-human world. If it is preferable for human moral motivation to extend to diverse non-human stakeholders, harnessing and orienting underlying intuitions towards the more-than-human world would be of great help, while acknowledging the fallibility of intuitions. In any event, given the current ecological crises, it appears critical that the expanded human impact should be accompanied by expanded moral concern.

## Data Availability

No datasets were generated or analysed during the current study.

## Footnotes

1 Our ethical viewpoint aligns with relational and intuitive conceptions of morality, thus focusing on moral processes beyond rational, conscious deliberation. This work contributes to the relational turn in ethics, sustainability research, and moral psychology, for instance, by elaborating self-transcendent moral emotions (Ruckert et al., 2025). From a relational perspective, ethics is a dynamic process constituted by affect-laden networks of interpersonal relationships that sustain social goods through the cultivation of affective bonds and the ongoing renegotiation of norms/expectations within interdependent systems. Morality is viewed to emerge from socioemotional processes and group dynamics, extending to relations with the more-than-human world. Unlike some other concepts related to the human-nature connection, NR emphasizes relationality, which in turn resonates with Haidt’s social intuitionism (introduced in more detail in the fourth section) in viewing felt connections and the formation and maintenance of social bonds as shaping moral judgements.

2 For further developments of biophilia as a systems biology-based, life-sustaining orientation, see Salmi 2025b.

3 These traditions align roughly with individualism versus sociocentrism, or WEIRD versus non-WEIRD contexts. However, collective-oriented cognitive styles are usually tuned to ingroup or societal concerns rather than broader biospheric interests. Greater collectivism does not necessarily enhance and may even constrain biocentric expansion.

4 For instance, identity can also be conceptualized as a broader, partly unconscious patchwork of identifications, indications of sameness or continuity, extending beyond explicit self-representations or self-referential thought.

5 This aligns with Weil and Murdoch’s moral epistemology, emphasizing non-egocentric, caring attention as central to ethics (Murdoch, 1970; Weil, 2021; Panizza, 2022; Aaltola, 2022). According to this realist view, morality involves perceiving and responding to these values, while egocentric selfhood obscures them. Individuals differ in their attribution of moral salience – some are blind, others attuned to the non-human world.

6 Even when groups on the surface share a stance against harming nature, their moral pathways often diverge. Anthropocentric intuitions treat nature as a set of *ecosystem services* where concern is triggered by resource depletion, whereas relational imperatives view the environment as a community of subjects. This divergence becomes clear when, e.g., studying intuitions regarding the extinction of *useless* species – those with no known medicinal, economic, or ecological benefit to humans (for background on cultural differences on nature-related issues, see f.ex. Medin & Bang, 2014).

7 Rottman et al. (2015) have suggested that the extension of individualizing moral concern will heavily rely on anthropomorphism, i.e., the misattribution of human characteristics to non-humans. Instead of conscious deliberation, such *perception of likeness* appears to stem primarily from embodied feelings (Servais, 2018). Conversely, for binding intuitions, such as sanctity, the attribution does not involve individual minds but, in turn, the projection of human purposes and meanings onto the system of nature (for more, see “promiscuous teleology,” Ojalehto et al., 2013). However, there are differing opinions on whether misattribution is problematic and whether conceiving non-human beings as persons should even be considered misattribution in the first place (Ruckert et al., 2025; Kohák, 1987; Harvey, 2005)​.
